# Efficacy of Rebamipide Instillation for Contact Lens Discomfort With Dry Eye

**DOI:** 10.1097/ICL.0000000000000438

**Published:** 2018-11-01

**Authors:** Tsutomu Igarashi, Maika Kobayashi, Chiemi Yaguchi, Chiaki Fujimoto, Hisaharu Suzuki, Hiroshi Takahashi

**Affiliations:** Department of Ophthalmology (T.I., M.K., C.Y., C.F., H.T.), Nippon Medical School, Tokyo, Japan; and Department of Ophthalmology (H.S.), Nippon Medical School Musashikosugi Hospital, Kawasaki City, Japan.

**Keywords:** Contact lens, Dry eye, Discomfort, OSDI, Rebamipide

## Abstract

**Objective:**

To examine the effects of rebamipide ophthalmic solution on the symptoms, signs, and cytokine concentrations in tear fluid among soft contact lens (SCL) wearers with Dry eye disease (DED).

**Methods:**

From November 2015 to June 2017, this open-label, single-arm study examined 40 eyes of 20 SCL wearers with DED who had been using daily disposable SCLs for >3 months (mean age, 30.0±8.33 years; range, 20–47 years). Signs, symptoms, and cytokine concentrations were assessed before and 4 weeks after starting 2% rebamipide ophthalmic solution 4 times/day. Dry eye disease was diagnosed according to: compromised tear dynamics (Schirmer test ≤5 mm or tear break-up time (TBUT) ≤5 sec); ocular surface abnormalities (positive vital staining with fluorescein or lissamine green); and presence of symptoms. Touch thresholds using a Cochet-Bonnet anesthesiometer were also determined for the cornea and conjunctivae. Symptoms were assessed using the 12-item Ocular Surface Disease Index questionnaire. Concentrations of cytokines in tear fluid were measured.

**Results:**

Significant improvements in signs were seen for TBUT, surface abnormalities, and touch thresholds. Ocular Surface Disease Index scores likewise improved significantly in all the 12 items. Of the cytokines measured, only interleukin-1β, interleukin-8, and monocyte chemotactic protein-1 were found in ≥60% of tear samples, with no significant differences in concentrations before and after rebamipide use.

**Conclusions:**

Rebamipide significantly improved all signs and symptoms in patients with DED who wore daily disposable SCLs. Rebamipide is effective for DED treatment with SCL wear.

Contact lenses (CLs), and soft CLs (SCLs) in particular, have become the principal means of refractive correction for hundreds of millions of people across the world.^1^ On the other hand, the number of patients with dry eye disease (DED) has been continuously increasing. In the United States, approximately 3.2 million women and 1.05 million men are estimated to have DED,^2^ whereas 30% of the population^3^ and one-third of office workers in Japan are believed to be affected by this disease.^4^ Thus, patients with DED wearing CLs are not at all rare, and 1 report suggested that 25% of CL users had to stop wearing CLs because of discomfort and dryness.^5^ Although artificial tears are often used in cases of eye discomfort or dryness, the effects are not always sufficient and patients sometimes have to stop wearing CLs.

Rebamipide ophthalmic solution (Mucosta ophthalmic suspension UD2%; Otsuka Pharmaceutical, Tokyo, Japan) has recently entered the market in Japan.^6^ Topical rebamipide has been shown to increase the number of mucin-containing goblet cells,^7^ and the utility of rebamipide ophthalmic solution for DED has been demonstrated in a phase III study,^8^ multicenter study^9^ and other recent studies, including our own report.^10–12^ The quinolinone derivative rebamipide was originally developed as a therapeutic agent for gastric ulcers through its activity in promoting mucin production in the gastric mucosa^13^ and its anti-inflammatory effects.^14^ Because dry eyes represent inflammation of the ocular surface,^15^ topical rebamipide should have both mucin-increasing and anti-inflammatory effects.

Currently, CL discomfort (CLD) is a substantial and burdensome problem experienced frequently by CL wearers.^16^ Contact lens discomfort is a condition characterized by episodes or persistent adverse ocular sensations related to CL use, either with or without visual disturbance, resulting from reduced compatibility between the CL and the ocular environment. Contact lens discomfort can lead to a discontinuation of CL wear.^17^ The prevalence of dryness and discomfort has been reported to range from approximately 30% up to 70% among CL users.^18,19^ As CLD is often associated with evaporative DED, the preferred treatment for CLD is similar to the treatment for evaporative DED,^20^ such as the use of preservative-free saline, hyaluronic acid instillation, or punctual plug. Little is currently known about the effects of rebamipide ophthalmic solution on CLD.

This study examined the effects of rebamipide ophthalmic solution on symptoms, signs, and cytokine concentrations in the tears of SCL wearers.

## MATERIALS AND METHODS

### Patients and Treatment

This study was a prospective open-label, single-arm study that followed the tenets of the Declaration of Helsinki and was approved by the Institutional Review Board at Nippon Medical School Hospital (approval number, 226025). Informed consent was obtained from all participants using the instructions that involved the adherence of ophthalmic administration, before any clinical evaluation was performed. Before subjects were enrolled, the study was registered at the Japanese University Hospital Medical Information Network Clinical Trials Registry (clinical trial identifier: UMIN000019738; Title name; Effect of rebamipide in SCL patients with dry eyes. Accessed November 11, 2015). The aim of this study was to examine the effects of rebamipide ophthalmic solution on the symptoms, signs, and cytokine concentrations in the tears of SCL wearers.

From November 21, 2015 to June 27, 2017, we examined 40 eyes of 20 SCL wearers with DED in the Department of Ophthalmology at Nippon Medical School Hospital and at Yaguchi Eye Clinic. We selected patients who had been using disposable SCLs (1-Day Acuvue or 1-Day Acuvue Moist; Johnson & Johnson, Tokyo, Japan) over 3 months. Patients who showed corneal erosion requiring treatment, had received a punctal plug, or had severe allergenic conjunctivitis were excluded. The mean (±SD) patient age was 30.0±8.33 years (range, 20–47 years). All patients were women. Signs and symptoms were assessed before treatments. All patients were prescribed 2% rebamipide ophthalmic solution 4 times/day, which was used with contact lenses on. Four weeks later, similar analyses were performed. We calculated the sample size according to our previous report^11^ and determined 20 patients at the beginning of this study, when the study was registered at the Japanese University Hospital Medical Information Network Clinical Trials Registry.

### Clinical Evaluation of Dry Eye

Dry eye was diagnosed according to the 2006 Japanese criteria for DED^21^ which contains three categories: compromised tear dynamics; ocular surface abnormalities; and existence of subjective symptoms. Compromised tear dynamics were determined using the Schirmer test and tear break-up time (TBUT). If either one of these tests yielded a positive result (Schirmer test, ≤5 mm; TBUT, ≤5 sec), tear dynamics were considered abnormal. An abnormal ocular surface was determined by positive vital dye staining using fluorescein and lissamine green. The degree of staining in the temporal and nasal conjunctiva and cornea, which were each divided into three parallel sections, was recorded and quantified on a score of 0 to 3. The maximum score (fluorescein staining score [FSS] or lissamine green staining score [LSS]) is nine for 1 eye. Both eyes were used to calculate the parameters. If either type of staining was positive, the ocular surface was considered abnormal. Patients with subjective symptoms, with abnormal tear dynamic, and ocular surface signs were considered to have definite dry eye, whereas patients with subjective symptoms where only one of the objective signs was positive were considered to have probable dry eye. In addition, touch thresholds were determined using a Cochet-Bonnet anesthesiometer with a 0- to 60-mm nylon monofilaments in 5-mm increments. The touch threshold was determined in the following five locations: center of the cornea; upper and lower palpebrae; and nasal and temporal bulbar conjunctivae.

### Symptom Score

Symptoms of DED were assessed using the Ocular Surface Disease Index (OSDI) questionnaire (Table 1).^22^ The 12 items of the OSDI questionnaire were graded on a scale of 0 to 4 as follows: 0, none of the time; 1, some of the time; 2, half of the time; 3, most of the time; and 4, all of the time. The total OSDI score was calculated sing the following formula: OSDI=(sum of scores for all questions answered 100)×100/(total number of questions answered)×4. The OSDI was scored on a scale of 0 to 100, with higher scores representing greater disability. The OSDI questionnaire comprised three subscales including ocular symptoms (Table 1, A–D), vision-related function (Table 1, E–I), and environmental triggers (Table 1, J–L). Subscale scores were calculated similarly, with only questions from each subscale used to generate that score.

**TABLE 1.**
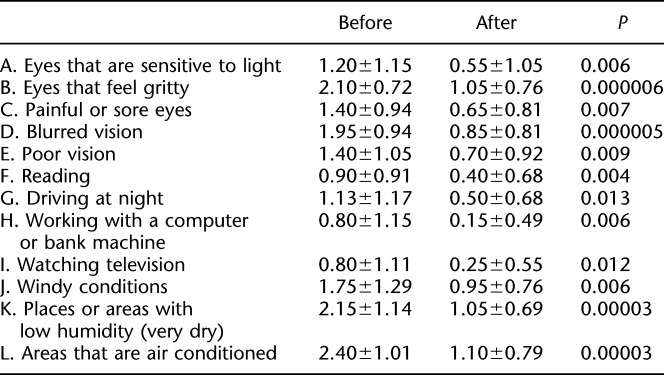
Ocular Surface Disease Index

### Tear Cytokine Concentration

Tears were collected from the filter paper used in the Schirmer tear production measuring strips (Showa Pharmaceutical, Tokyo, Japan). The filter paper was stored in an Eppendorf tube (Eppendorf, Tokyo, Japan) at −20°C until analysis. After the filter paper was thawed at room temperature, the filter paper was cut at 10 mm length from the end, then tear fluid was eluted using a 100-μL elution solution consisting of 0.01 M phosphate-buffered solution with 0.5 M NaCl, 0.5% Tween 20, and 0.5% bovine serum albumin for 3 hr, as previously reported.^23^ Concentrations of the following 10 cytokines were measured using the Bio-Plex Pro Human Cytokine 10-plex Assay (Bio-Rad, Hercules, CA) in accordance with the instructions from the manufacturer: interleukin (IL)-1β, IL-4, IL-6, IL-8, IL-10, IL-12 (p70), IL-17A, interferon (IFN)-γ, monocyte chemotactic protein (MCP)-1, and tumor necrosis factor (TNF)-α. Standard curves and concentrations of cytokines were determined using the Bio-Plex 200 system (Life Science, Hercules, CA) and Bio-Plex Manager version 6.1 software (Life Science). The cytokine concentrations were compared between before and at 4 weeks after the start of rebamipide administration. To adjust differences in each tear volume, data were corrected using the score of Schirmer test at each collected time.

### Statistical Analysis

Statistical analyses were performed by paired *t* test for objective signs (TBUT, FSS, LSS, Schirmer test, corneal sensitivity, and conjunctival sensitivity [CS]) and tear cytokines for each eye, and by the Wilcoxon *t* test for subjective symptoms (OSDI) for each eye, using Stat Flex version 6 software (Artec, Osaka, Japan).

## RESULTS

### Patients

We enrolled 28 SCL users. Eight patients did not fulfill the Japanese criteria for DED. Twenty patients were assigned and allocated to 2% rebamipide ophthalmic solution. Twenty patients completed the follow-up and were analyzed in all examinations.

### Objective Parameters

**FIG. 1.**
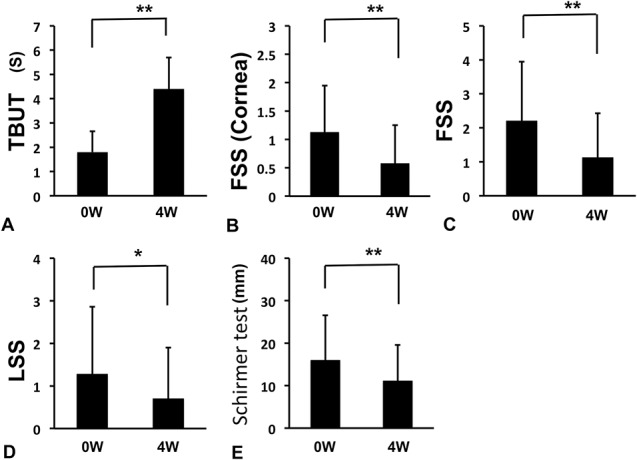
Changes in objective signs before and after the 4-week treatment. (A) Tear break-up time (TBUT). (B) Fluorescein staining score (FSS) in cornea. (C) Total FSS. (D) Lissamine green staining score (LSS). (E) Schirmer test I. **P<0.01, *P<0.05, paired *t* test.

**FIG. 2.**
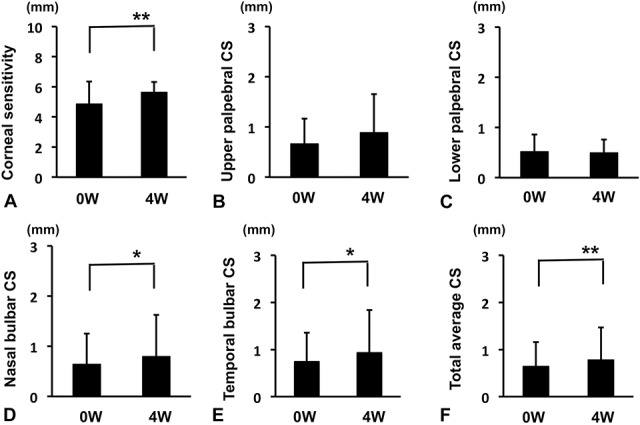
Changes in corneal and conjunctival sensitivity (CS) before and after the 4-week treatment. (A) Corneal sensitivity. (B) Upper palpebral CS. (C) Lower palpebral CS. (D) Nasal bulbar CS. (E) Temporal bulbar CS. (F) Total average CS. **P<0.01, *P<0.05, paired *t* test.

Objective parameters before and at 4 weeks after starting rebamipide administration were compared (Figs. 1 and 2). Significant improvements were found in TBUT (Fig. 1A; 1.8±0.85 sec to 4.4±1.3 sec, *P*<0.01), corneal FSS (Fig. 1B; 1.13±0.82 to 0.58±0.68, *P*<0.01), total FSS (Fig. 1C; 2.2±1.74 to 1.13±1.3, *P*<0.01), and LSS (Fig. 1D; 1.28±1.58 to 0.7±0.7, *P*<0.05). On the other hand, Schirmer test decreased significantly from 15.98±10.57 mm to 11.08±8.5 mm (Fig. 1E; *P*<0.01). Corneal sensitivity, nasal bulbar CS, and temporal bulbar CS increased significantly from 4.88±1.48 mm to 5.65±0.66 mm (Fig. 2A; *P*<0.01), from 0.64±0.61 mm to 0.8±0.82 mm (Fig. 2D; *P*<0.05), and from 0.75 to 0.94±0.9 mm (Fig. 2E; *P*<0.05), whereas upper and lower palpebral CS did not change significantly from 0.67±0.5 mm to 0.89±0.76 mm (Fig. 2B) and from 0.52±0.34 mm to 0.52±0.34 mm (Fig. 2C). The change in total average CS was significant (Fig. 2F; 0.65±0.51 mm to 0.78±0.69 mm, *P*<0.01).

### Ocular Surface Disease Index Score

**FIG. 3.**
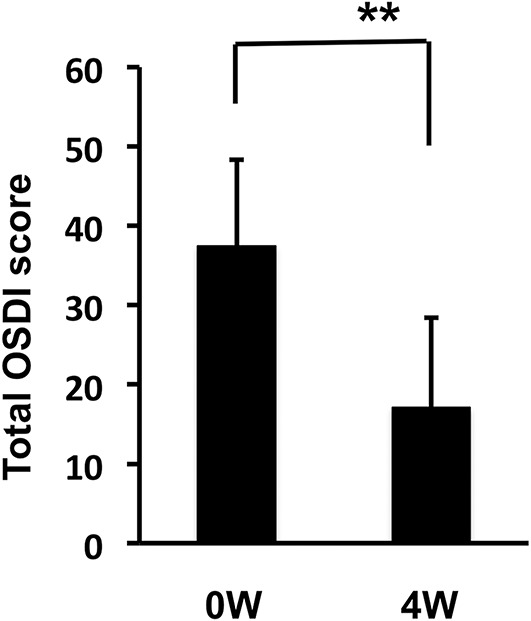
Changes in the total OSDI score before and after the 4-week treatment. Total OSDI scores at 4 weeks after starting rebamipide administration were significantly improved. **P<0.01, Wilcoxon *t* test.

**FIG. 4.**
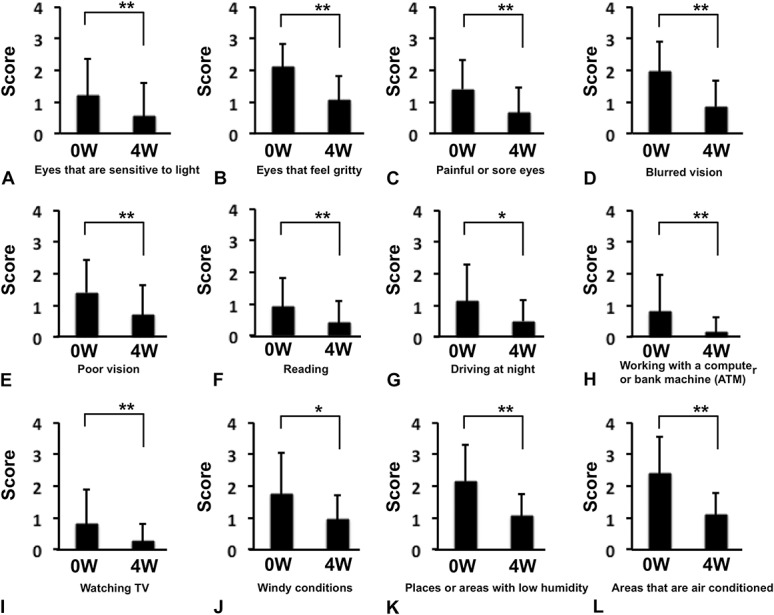
The 12 items in the OSDI. (A) Eyes that are sensitive to light. (B) Eyes that feel gritty. (C) Painful or sore eyes. (D) Blurred vision. (E) Poor vision. (F) Reading. (G) Driving at night. (H) Working with a computer or bank machine (automated teller machine [ATM]). (I) Watching television. (J) Windy conditions. (K) Places or areas with low humidity (very dry). (L) Areas that are air conditioned. **P<0.01, *P<0.05, Wilcoxon *t* test.

Ocular Surface Disease Index score after 4 weeks of rebamipide use significantly improved compared with pretreatment (Fig. 3; 37.58±10.9 to 17.08±11.33, *P*<0.01). Significant improvements were observed in all the 12 items (Table 1 and Fig. 4).

### Tear Cytokine Concentrations

**FIG. 5.**
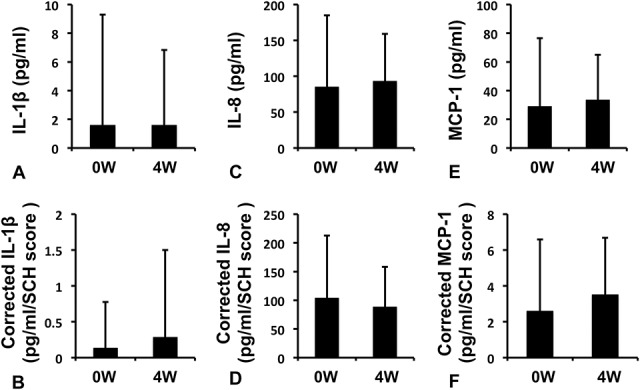
Changes in the tear cytokine concentrations before and after the 4-week treatment. Tear cytokine concentrations were compared between before and after rebamipide administration. To adjust differences in each tear volume, data were corrected using the score of Schirmer test (corrected value). (A) Interleukin (IL)-1β. (B) Corrected IL-1β. (C) IL-8. (D) Corrected IL-8. (E) Monocyte chemotactic protein (MCP)-1. (F) Corrected MCP-1. No cytokines showed significant decreases (paired *t* test).

Of the 10 cytokines measured (IL-1β, IL-4, IL-6, IL-8, IL-10, IL-12 (p70), IL-17A, IFN-γ, MCP-1, and TNF-α), only three cytokines (IL-1β, IL-8, and MCP-1) were found in ≥60% of tear samples (Table 2). Comparisons of the concentrations of these three cytokines before and after rebamipide instillation showed no significant differences (Figs. 5A,C,E). Also, Schirmer score–corrected cytokine concentrations showed no significant differences (Figs. 5B,D,F).

**TABLE 2.**
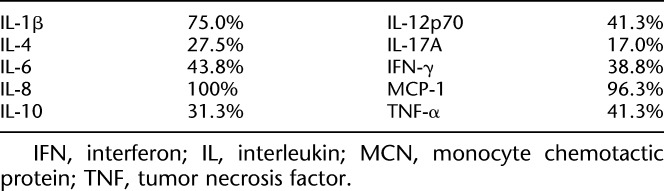
Percentage of Measurable Tear Samples

## DISCUSSION

Rebamipide was originally developed for the treatment of gastric ulcers^24,25^ due to the effective increase in mucin production by the gastric mucosa.^26^ Mucin is also an important lubricant for the ocular surface and helps to stabilize the tear film.^27,28^ Rebamipide has therefore also been marketed for instillation, and many recent studies, including our own,^11^ have reported the efficacy of this agent in the treatment of DED.^9,29,30^ Our previous study found that rebamipide significantly improved TBUT and FSS, but resulted in no changes in Schirmer test scores.^11^ Kinoshita S, et al. reported significant improvements in TBUT, FSS, and LSS, but also no changes in Schirmer test scores.^9^ On the other hand, Igarashi A, et al. reported significant improvements in TBUT, FSS, and Schirmer test scores.^31^

This study investigated the effectiveness of rebamipide for SCL users with DED, and similar to the effectiveness for usual DED, improvements were seen in all objective parameters. Interestingly, corneal sensitivity improved and Schirmer test score decreased. Decreased corneal sensitivity in CL users has been reported,^32,33^ and this decreased corneal sensitivity may increase the risk of corneal infection. This improvement is thus highly significant. A decrease in Schirmer score seemed to disagree with other favorable effects of rebamipide in the study, however, the score was still within the normal range suggesting that the excessive tearing was improved by the eye drops. We speculated that some effects by the rebamipide on corneal sensitivity might result in decrease in Schirmer score. Soft CL wearers often have mild corneal erosions, which can cause mild chronic pain stimulus. Rebamipide instillation improved the condition and stabilized the trigeminal nerve, and thus reduced excessive tearing, which resulted in decrease in Schirmer score.

For all parameters of the OSDI^22^ scores, subjective symptoms improved with rebamipide. Analysis of the three subscales including ocular symptoms (Table 1, A–D), vision-related function (Table 1, E–I), and environmental triggers (Table 1, J–L) showed the greatest improvement in environmental triggers, which contributed to the improvement in subjective symptoms. An interesting association between OSDI scores and pre-CL tear film kinetics has recently been reported.^34^ CL use can segmentalize tear film, decrease tear meniscus height,^35^ and increase tear film evaporation.^36^ According to that study,^34^ patients with high OSDI scores (i.e., severe symptoms) develop instability of the pre-CL tear film kinetics. Improved tear meniscus dynamics on rebamipide instillation^37^ can therefore help alleviate subjective symptoms.

This study compared tear cytokine concentrations before and at 4 weeks after rebamipide administration. Schirmer test strips were cut at 10 mm, and proteins in the test strip were extracted and used for multiplex bead analysis (Bio-Plex). Increased concentrations of IL-1β, IL-5, IL-6, IL-8, IFN-γ, and TNF-α have been reported in patients with DED.^38^ Meanwhile, among CL users, increased levels of IL-6, IL-8, and TNF-α have been reported.^39,40^ In our study, the only cytokines that could be measured in ≥60% of samples were IL-1β, IL-8, and MCP-1 (Table 2). Although the levels of these proteins tended to decrease, these values, together with the Schirmer test–corrected values, did not differ significantly between before and after rebamipide instillation. One reason may have been the large variations in measured values for all parameters. Therefore, whether tear cytokine concentrations can be improved in CL users will require further investigation.

A weakness of the current study is the limited number of subjects, which composed only female participants. Although we did not intentionally select female patients in the study, eventually only female participants were examined. Also, the study lacked the control. Because this research was a pilot study to examine the effect of rebamipide eye drop in SCL wearers, it was designed as a prospective open-label, single-arm study. These conditions could invite a bias in the results and decrease reliability of the conclusions. A randomized comparative prospective study should be performed in the future.

In conclusion, rebamipide instillation significantly improved all symptoms and signs in patients with DED who wore daily disposable CLs. Rebamipide is effective for DED treatment with SCL wear.
